# Tunable holographic metasurfaces for augmented and virtual reality

**DOI:** 10.1515/nanoph-2024-0734

**Published:** 2025-02-17

**Authors:** Akeshi Aththanayake, Andrew Lininger, Cataldo Strangi, Mark A. Griswold, Giuseppe Strangi

**Affiliations:** Department of Physics, 2546 Case Western Reserve University, 2076 Adelbert Rd., Cleveland, OH 44106, USA; Department of Radiology, Interactive Commons, 2546 Case Western Reserve University, 10900 Euclid Ave., Cleveland, OH 44106, USA; University of Calabria and CNR – Institute of Nanotechnology, Rende, CS, Italy

**Keywords:** metasurface, metamaterials, augmented reality, virtual reality, tunable metasurface

## Abstract

Augmented and virtual reality (AR/VR) is transforming how humans interact with technology in a wide range of fields and industries, from healthcare and education to entertainment. However, current device limitations have impeded wider integration. Tunable holographic metasurfaces represent a promising platform for revolutionizing AR/VR devices by precisely controlling light at the subwavelength scale. This article examines current challenges and opportunities from both the AR/VR and holographic metamaterial perspectives and explores how improvements to state-of-the-art approaches can address these challenges. In particular, we propose a focus on easily manufacturable and broadly electrically tunable metasurface technologies including liquid crystal integration and excitonic tuning in 2D materials. Advanced metasurface optimization techniques including machine learning will also be crucial for exploring the large design space.

## 1 Introduction

Augmented and virtual reality (AR/VR) devices are normally head-worn displays that project light directly into the eyes to simulate the appearance of 3D objects in front of the wearer. These devices will generally fall into one of two categories: AR headsets that use transparent waveguides to allow the wearer to directly see the physical world as well as simulated objects, and VR headsets that use opaque displays to simulate a full virtual world. Both devices require precise control of light close to the eyes, low weight, and low power consumption so that they can be worn on the head comfortably and without excess heating [1].

**Figure 1:**
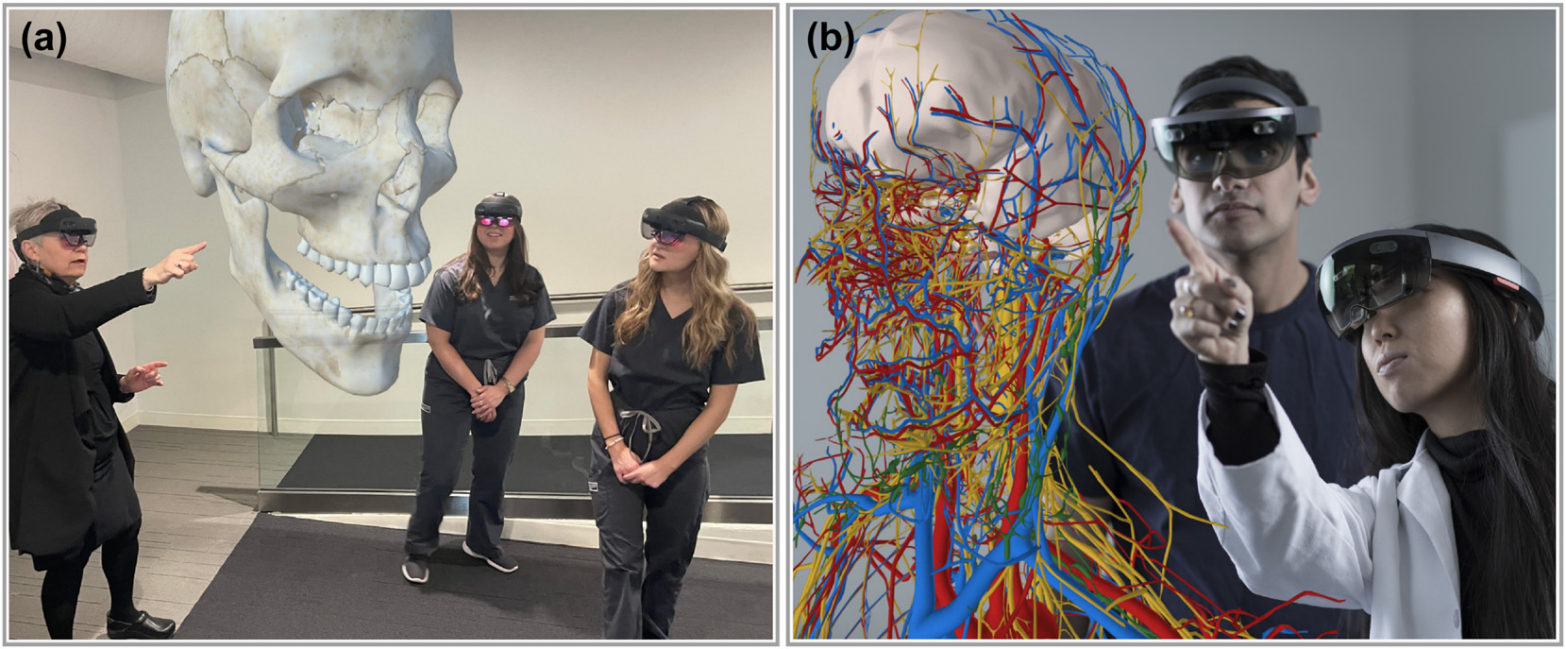
Example applications of current AR/VR devices in education and medicine. (a) Dr. T. Roma Jasinevicius from Case Western Reserve University teaches dental anatomy to first- and second-year students using AR technology. (b) Students using AR technology to visualize complex anatomical structures of the head and neck in real time. (Courtesy of Interactive Commons, Case Western Reserve University, Cleveland, Ohio.)

The current market for these devices is significant, with $7 billion in revenue globally in 2023 and an annual growth rate of up to 50 % [2]. While many applications have focused on gaming, the last decade has also shown a multitude of examples where AR/VR technology is used in manufacturing, education, and medicine, demonstrating a clear need for advanced 3D display technologies. For example, Lockheed Martin has used AR/VR headsets in the manufacturing of the Orion spacecraft. Using an AR/VR headset to show the exact position of fasteners on the spacecraft in AR resulted in a 90 % reduction in assembly time and a complete elimination of assembly errors [3]. AR/VR technology has also been shown to reduce the time it takes for medical students to learn human anatomy by a factor of two while maintaining the same level of performance on benchmark exams [4], see Figure 1. In the clinical world, these technologies can reduce the time to perform medical procedures by nearly a factor of two [5]. AR/VR technologies are even making an impact on our understanding of the structure of the brain by allowing experts to form a consensus around complicated 3D structures [6]. These are just a few examples that demonstrate the immense potential of AR/VR technologies to drive innovation across multiple fields. However, the future adoption and application of AR/VR devices hinges on improving wearability, accessibility, and usability, which is accomplished by decreasing hardware cost, weight, and power consumption.

Holographic metamaterials merge holography with subwavelength engineering to dynamically control light, providing essential tools for AR/VR near-eye displays and enhancing the immersive quality of AR/VR by blending virtual content seamlessly with real-world perspectives [7]. Unlike traditional optics, which rely on bulky components, these materials use nanoscale fabricated “meta-atoms” to manipulate light’s phase, amplitude, and polarization on a compact surface. A high degree of control is possible, with achievable full 2*π* phase delay, allowing for lightweight designs that can reconstruct 3D images and perform complex optical functions [8], [9], [10], [11]. This control is particularly valuable in holography, where realistic 3D images are created by reconstructed light fields from a complicated phase surface, and highly local phase control is critical. In AR/VR applications, this approach allows devices to match virtual images with real-world views, providing immersive, adaptable experiences [12].

Scattering or diffractive metasurfaces will be important for next generation holographic displays, controlling light precisely through the interaction with nanostructured materials. The scale of the structuring means that the device thickness can be drastically decreased, reducing form factor. This ensures clarity in virtual images while reducing the weight and bulk associated with traditional optics – crucial for wearable AR/VR displays [13]. Advances by Capasso and colleagues in flat optics have established ultra-thin, high numerical aperture (NA) meta-lenses, feasible for integrating in wearable AR/VR displays for enhanced field-of-view (FOV) and reduced form factor [14], [15], [16]. In particular, transmissive dielectric metasurfaces are important for their high diffraction efficiency and low optical losses.

Beyond integrating holographic metasurfaces, AR/VR applications can be further advanced by tunable metasurfaces, enabling a dynamic response from a single optical device. To date, the most studied application has been dynamic focal adjustments in tunable metalenses [10], [17], [18]. These devices are well-poised for implementation in holographic displays, where depth variations enhance 3D viewing comfort. They can adjust to user gaze or environmental conditions, offering seamless focal transitions for natural AR/VR interactions [19]. As metasurface design has advanced, tunable holographic metamaterials have evolved to enable complex optical functions on a single surface, tailored to produce specific conditions such as different wavelengths responses or desired focal corrections [20]. This multifunctionality allows scalable, compact designs without added components.

**Figure 2:**
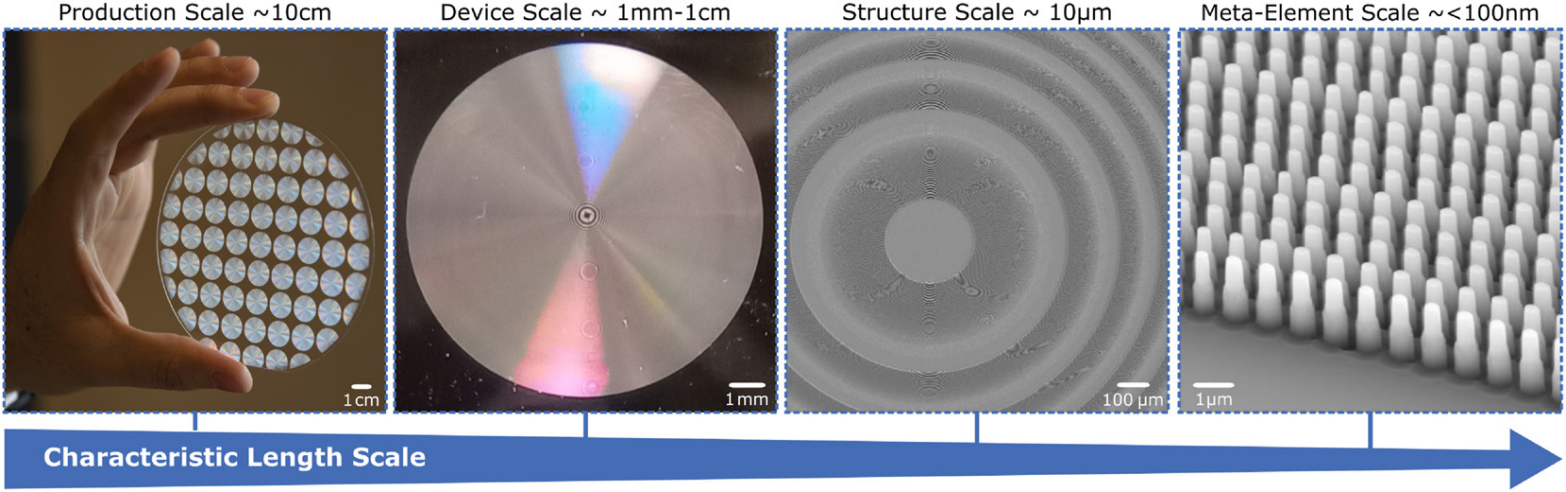
Metasurfaces across scales. Metasurfaces require attention to design and fabrication across a large range of length scales from the production (∼10 cm), to the device (∼1 mm–1 cm), structural (∼10 μm), and meta-element (∼<100 nm) scales. The metasurface shown in this figure is described in detail elsewhere [25], and images are adapted with permission [26]. Copyright 2020, Proceedings of the National Academy of Sciences.

One of the most critical aspects of metasurface implementation is the design and optimization of the subwavelength scale structure, to take advantage of the tremendous design space [8], [21], [22]. Metamaterials require strict attention to optimization and fabrication across a staggering range of length scales, from centimeters to nanometers, see Figure 2. As applications advance from relatively “simple” applications such as varifocal lenses where the phase conditions are broadly known to tunable holograms and free-form metasurfaces with hundreds of millions of geometrical degrees of freedom, the design complexity increases substantially. Optimization tools and precise manufacturing are key to meeting the demands for AR/VR technology, with modern approaches such as machine learning becoming increasingly important [8], [23], [24].

## 2 Challenges and opportunities

Metasurfaces, and especially tunable holographic metasurfaces, are well-positioned for large-scale integration into AR/VR devices, enabling next-generation systems. The challenges faced by current AR/VR approaches present opportunities for significant improvements with metasurface technologies. However, future implementations will require targeted advances in tunable metasurface technologies to overcome key technological limitations.

### 2.1 AR/VR devices

The potential market and applications range for AR/VR technologies is huge. The field is already showing significant uses for AR/VR technologies across industry, medicine, education, entertainment, and beyond. In fact, once the form factor of the complete AR/VR headset becomes small enough to resemble a normal pair of glasses, many tech leaders believe that AR/VR technology could replace mobile phones as the primary way we interact with the digital world [27].

To achieve this level of integration, the overall system performance will need to significantly improve over today’s generation of devices. Future displays must have a large enough FOV to be useful in everyday life; the human eye can observe up to 160° angles, including eye roll [28]. However, maintaining the display resolution for a larger field of vision requires a greater number of pixels to be calculated and drawn per frame. Additionally, the calculation and device actuation must operate at a minimum frame rate of ∼120 Hz to reduce adverse reactions [29]. This results in more computation with ensuing heating and power consumption. Further improvements in image quality, resolution, and precise control of device focal parameters, such as dynamic focus correction and binocular alignment, are important for enhancing usability, reducing visual strain, and improving depth perception [30], [31]. Many of these improvements, such as decreasing the form factor, are targeted at increased user comfort as well as device performance.

The entire AR/VR field requires development across all of these areas. On the computational side, advancements in processor efficiency and battery technology offer promising solutions to address the computational and power challenges above. However, many aspects are fundamentally linked to the device optical hardware and elements. In these cases, advanced optical technologies, including holographic metasurfaces, are a clear pathway for device improvement. Now is the time to fully develop and integrate new AR/VR display technologies for the next generation of applications.

### 2.2 Metasurfaces and tunability

AR/VR devices can significantly benefit from the integration of metasurface technologies, including both globally and locally tunable systems. Tunable, or active, metasurfaces offer all the benefits of metasurface technologies including extremely fine light manipulation and small form factor, while letting the system take on a range of optical states [32], [33], [34]. These systems offer an alternative approach for dynamic wavefront control over traditional AR/VR technologies by allowing the system to achieve the wavefront conditions of multiple optical systems in a single device package. Future integrations have the potential to reduce form factor and improve resolution, optical functionality, and switching speed in AR/VR devices. Although this approach is conceptually simple, there are significant hurdles to practical device implementations.

Several key improvements are essential for advancing holographic metasurfaces for AR/VR applications: increasing fidelity in a commercially viable nanopatterned surface, broadening the spectral response range to include the entire visible spectrum, and implementing the optical properties over the entire field of view. Fidelity encompasses both the fabrication quality, which is nontrivial in commercially viable devices, and the ability to manipulate the wavefront at the desired resolution [35], [36]. Although many fabrication techniques can perform at the requisite resolution, most have extremely low throughput and are not applicable for mass-produced devices. Significant additional hurdles, including real-world durability and environmental impact, remain severely underexplored and must be addressed for future devices.

Metasurfaces produce extraordinary optical responses, but demonstrated devices are typically limited to specific spatial angles and frequency ranges. Although recent improvements in multichromatic devices are promising, limits exist, and the requirements of full-visible range processing make AR/VR applications particularly difficult [16], [37]. Off-axis engineering is also difficult for metasurfaces due to off-axis aberrations (especially coma) arising from the phase-angle dependence [38].

Another key area for advancement is improving the addressability, or enabling local control of the metasurface at the micro or nanoscale. Decreasing the length scale of the tunable response effectively increases the pixel pitch and density, leading to more precise wavefront control. Ideally, this control would extend to the level of an individual meta-element, though this remains practically unfeasible. Global or large-scale tunable controls can only access a limited number of device states and are fundamentally limited in application. In principle, a holographic metasurface allowing for full 2*π* phase control over the wavefront in an AR/VR device may require many millions of distinct tunable states. It should be noted that the operating principle of wavefront control is similar to a spatial light modulator, although the quality and form factor enabled by metasurface devices is in principle much greater [39]. Although challenges remain, a number of recent studies have made tremendous progress in shortening the gap between holographic metasurfaces and commercially viable AR/VR device implementations.

## 3 Key metasurface technologies

Tunable metasurfaces offer a highly versatile platform for manipulating light, enabling dynamic control over its propagation by the phase, amplitude, polarization, and spectral response, among others [34], [40], [41]. These devices are a central focus of current photonics research [8]. Dynamic tunability in metasurfaces is achieved either by designing nanostructures that respond to external stimuli, or incorporating active materials to dynamically control the local environment. Common stimuli include electric and light fields [42]; active materials include liquid crystals, phase-change materials (PCMs), and electronically tunable materials such as 2D transition metal dichalcogenides (TMDs). By leveraging these mechanisms, tunable metasurfaces can enable real-time control over light propagation, essential for varifocal lenses and dynamic holographic displays in AR/VR systems [43]. The most common categories of metasurface fabrication materials and strategies for incorporating tunability are listed in Table 1.

**Table 1:** Comparison of the (top) most common categories of metasurface fabrication materials and (bottom) strategies and materials for implementing tunability. RI refers to the material refractive index.

Material	Characteristics	Advantages	Limitations
**Material classes for metasurface fabrication**			
Polymers	Flexible, low RI	Low-cost, scalable	Low thermal and mechanical stability, distortions
Low index dielectrics	RI <2 , low optical losses	High transparency	Limited phase control, weak confinement, scalability
High index dielectrics	RI >2 , low optical losses	High efficiency, low optical losses	Scalability
Metals	Strong plasmonic resonances	High efficiency optical response	Intrinsic material losses and optical absorption
**Strategies and materials for implementing tunability**			
2D materials	Atomically thin, excitonic resonances	Addressability, response time, electrically tunable	Large scale synthesis and integration
Liquid crystals	Anisotropic refractive index, liquid material	Established technology, electrically tunable	Power requirements, thermal instability
Phase-change materials	Reversible amorphous-crystallization transition	High RI contrast, nonvolatile switching	Durability, power demands, fabricability

**Figure 3:**
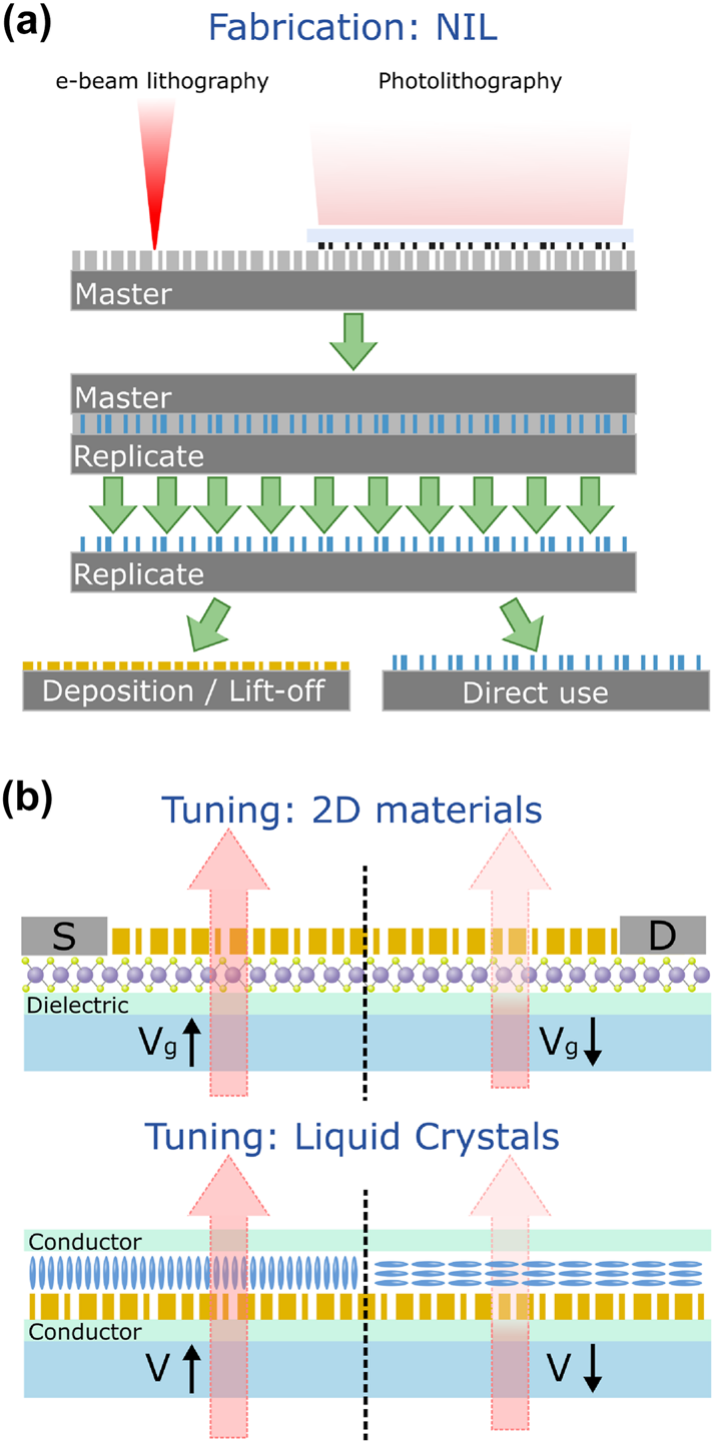
Strategies for fabrication and tuning of AR/VR metasurface devices. (a) Nanoimprint lithography (NIL) utilizes a single nanopatterned mold (master) to produce many replica nanoscale surfaces with an inverse structure, which can be used in inverse masking processes or directly as polymeric metasurfaces. Since the initial lithography is only performed once, the technique highly scalable and cost-effective. (b) Some of the most promising schemes for tuning metasurface devices are (top) electronic (gate voltage Vg) tuning of the light–matter interaction in 2D material coupled metasurface devices, including transition metal dichalcogenides (MoS_2_ shown), and (bottom) liquid crystal devices (with applied electric field at voltage V) integrated with metasurfaces to control the ambient refractive index. The liquid crystal is shown to reorient in the applied electric field.

Focusing first on device fabrication, deep-UV lithography and nanoimprint lithography (NIL) are among the most promising commercially viable nanofabrication techniques [25], [44], [45], [46], [47]. In particular, NIL should be highlighted as a cost-effective method for creating nanoscale patterns in macroscale objects, covering the range of fabrication length scales and enabling scalable production of metasurfaces. The technique can serve as a replacement for traditional e-beam and photolithography processes, as illustrated in Figure 3(a). The patterned material is naturally polymer-based and can either be coated – using for instance an inverse masking and lift-off process – to create a metasurface in a different material or used directly as a dielectric polymer metasurface. Although most nanopatternable polymers such as PDMS [48] have a low refractive index, polymers can be desirable as metasurface materials in some applications due to their flexibility, mechanical properties, potentially high optical quality, transparency, and low-cost fabrication. Additionally, the growth of additive manufacturing techniques, such as two-photon polymerization lithography, could be useful for small-batch samples [49], [50]. Of depositable materials, dielectrics such as TiO_2_ and Si_3_N_4_ are the most desirable for AR/VR devices due to their high refractive indices in the visible range (*n* ∼ 2) and low optical losses compared with plasmonic materials. These properties engender high clarity and energy efficiency in metasurface devices [28]. Ultimately, when choosing a material, durability and compatibility with the chosen tuning method are paramount.

Although there are now a number of strategies for enabling tunability in holographic metasurfaces, we propose that integrating liquid crystals or 2D materials such as transition metal dichalcogenides (TMDs) are the most promising avenues for meeting the challenges of AR/VR applications. These are illustrated in Figure 3(b).

Liquid crystals exhibit a highly responsive reorientation effect in electric fields, among other tunable parameters, enabling a degree of local refractive index modulation within the metasurface [51]. A number of recent studies have utilized liquid crystals for metasurface applications such as varifocal lenses [17], [18], [52], and the physics and control technology are well established [53], [54]. Additionally, the tuning rate or switching speed far exceeds the required AR/VR refresh rate [55]. Despite their responsiveness and high degree of tunability, the drawbacks of utilizing liquid crystals as an active medium include higher power requirements for maintaining orientation, limitations on the local control due to long-range ordering, and thermal instabilities [56]. The issue of long interaction lengths becomes more significant as device dimensions are scaled down, and while it can be mitigated through adjustments to molecular chemistry, it cannot be fully eliminated. For these reasons, we propose that achieving further advancements in AR/VR devices will necessitate the development of more precise and tunable control mechanisms. Nevertheless, this technology warrants continued investigation due to its significant potential for near-term device improvements and ease of implementation.

Another class of materials, which have garnered much attention for optical tunability over the last few years, are 2D materials such as TMDs, with the most common being MoS_2_ and WS_2_. These materials offer unique excitonic properties, enabling optical tuning through applied electrical field gating [57], [58], as well as optical modulation [59]. In electronic gating schemes, interaction between the injected charge and excitonic modes can lead to local refractive index changes in the visible spectrum [60], [61], [62], [63], [64]. Integration with metasurfaces engender fast, solid-state tunable devices for total wavefront control, such as mirrors [65], lenses [66], and beam scattering control at high modulation frequencies [67]. Although these materials show promise for ultra-thin, highly tunable AR/VR holographic metasurfaces, research remains in the early stages. Significant additional effort is required to develop and understand systems at the level of AR/VR device integration. For fabrication, mechanical exfoliation is the most reproducible and widely available process. However, this is not currently a commercially scalable process, limiting the large-scale production of TMDs. Additionally, the long-term stability of these devices is not well understood, particularly when considering degradation mechanisms such as photo-induced spontaneous oxidation [68].

A key consideration when implementing a tunable mechanism is the external stimuli or stressors necessary to enable the dynamic response in the material, since this can have a large impact upon the device form factor, implementation, and usage [34], [69]. Electrical and optical stimuli are the most important for highly tunable, compact, and portable implementations, although other stimuli such as temperature, chemical, and mechanical exist [70]. Electrical tuning is preferred for its speed, reversibility, and integrability. Also, electronic control processes are well established and integrated with eminently electronic AR/VR devices. The main strategy for increasing the local control in liquid crystal integrated devices is interdigitation and miniaturization of contacts, similar to spatial light modulator technologies, which can be accomplished with traditional or CMOS techniques [71], [72]. In TMD materials, local control over the excitonic properties can be established through gating techniques [49], [57], [61], [73], where a blueprint for establishing fine-scale electronic control already exists in the semiconductor industry. In addition, all-optical control processes are interesting since in principle they do not require a direct contact method with the metasurface device and can achieve smaller form factor and exceptional switching speeds in 2D excitonic materials [74]. Although the tunable response expands the functional scope of metasurfaces, balancing speed, precision, and durability remains an area of ongoing research.

### 3.1 Current approaches

The integration of metasurfaces into AR/VR devices has seen significant advancements in recent years. Ultra-thin, flat optics enable reduced form factor, improved optical performance, and the incorporation of tunable functionalities. In general, their development has focused on specific use cases, such as meta-holograms [75], meta-couplers, and tunable metalenses.

Recent developments in near-eye displays for AR/VR leverage phase-only holographic projection based on Fresnel holography and double-phase amplitude encoding. These systems, utilizing spatial light modulators (SLMs) for precise control of light amplitude and phase, enable full-color, high-contrast, and high-resolution holograms with true per-pixel focal adjustments. GPU-accelerated computation allows real-time hologram generation, with integrated focus, aberration correction, and vision correction models addressing optical defects. These compact, eyeglasses-like displays achieve wide fields of view (up to 80°), showcasing the potential for revolutionary AR/VR experiences [76]. Phase-change metasurfaces have been employed to create rewritable holographic displays, where holograms can be reprogrammed by altering the material’s crystalline state using thermal or optical stimuli [77]. To achieve tunability in meta-holograms, materials like phase-change compounds and liquid crystals are commonly used to dynamically switch or modify holographic patterns. This capability enables applications such as personalized or real-time holographic interfaces in AR [78].

Meta-couplers serve as efficient interfaces between light sources and optical waveguides in AR/VR systems. They direct light from a display engine into waveguides, enabling compact and high-efficiency light propagation. Recent advancements have resulted in meta-couplers with greater efficiency, broader operational bandwidths, and reduced optical losses [79]. Tunable meta-couplers further enhance functionality by dynamically redirecting light to optimize coupling efficiency across various wavelengths or polarization states, crucial for full-color AR/VR displays. A recent demonstration utilized dielectric metasurfaces in meta-couplers that dynamically adjust coupling efficiency using external electric fields, providing superior brightness control in AR waveguide displays [80].

Moreover, metasurfaces have been shown to replace or enhance traditional optical components in AR/VR devices, including lenses and mirrors, offering ultra-thin solutions for wavefront control. Their ability to dynamically modify optical properties, including phase, polarization, and amplitude, has been pivotal to their integration in AR/VR technologies [14], [28], [81], [82]. In a recent work by Li et al. from the Capasso group, an RGB-achromatic metalens was developed to address the challenges of chromatic aberration and large form-factor optics in near-eye display systems for AR/VR applications [16]. The metasurface utilized dispersion engineered TiO_2_ meta-atoms and zone interference design principles, optimizing local phase, group delay, and group delay dispersion (GD-GDD) to produce 2-mm-diameter lenses with Numerical Aperture (NA) = 0.7 and 0.3, respectively. This approach allows constructive interference over a wide spectral range, achieving diffraction-limited achromatic focusing across the red, green, and blue spectral bands with Strehl ratio ≥97 % and focusing efficiencies in the range of 10 %–16 % at different wavelengths. This degree of focusing quality significantly exceeds the capabilities of traditional refractive lenses. Through use of a doublet configuration, the FOV at high resolution was extended to ∼50° . Beyond the extraordinary optical performance, compactification of the AR/VR optical pathway afforded by the metalens integration allowed the researchers to overcome the limitations of traditional bulky eyepieces, providing enhanced contrast and minimizing both monochromatic and chromatic aberrations. This was demonstrated through a fiber scanning near-eye display system, enabling real-time image projection with minimal pixel size. In this example, metalens integration combines compactness, extremely high resolution, and performance over a wide color gamut, demonstrating a pathway for future devices. The addition of tunable elements has the potential to further improve this technology. For example, dielectric metasurfaces combined with liquid crystals have been used to develop varifocal lenses for AR glasses, achieving rapid focal length adjustments with minimal energy consumption [83].

## 4 Discussion

**Figure 4:**
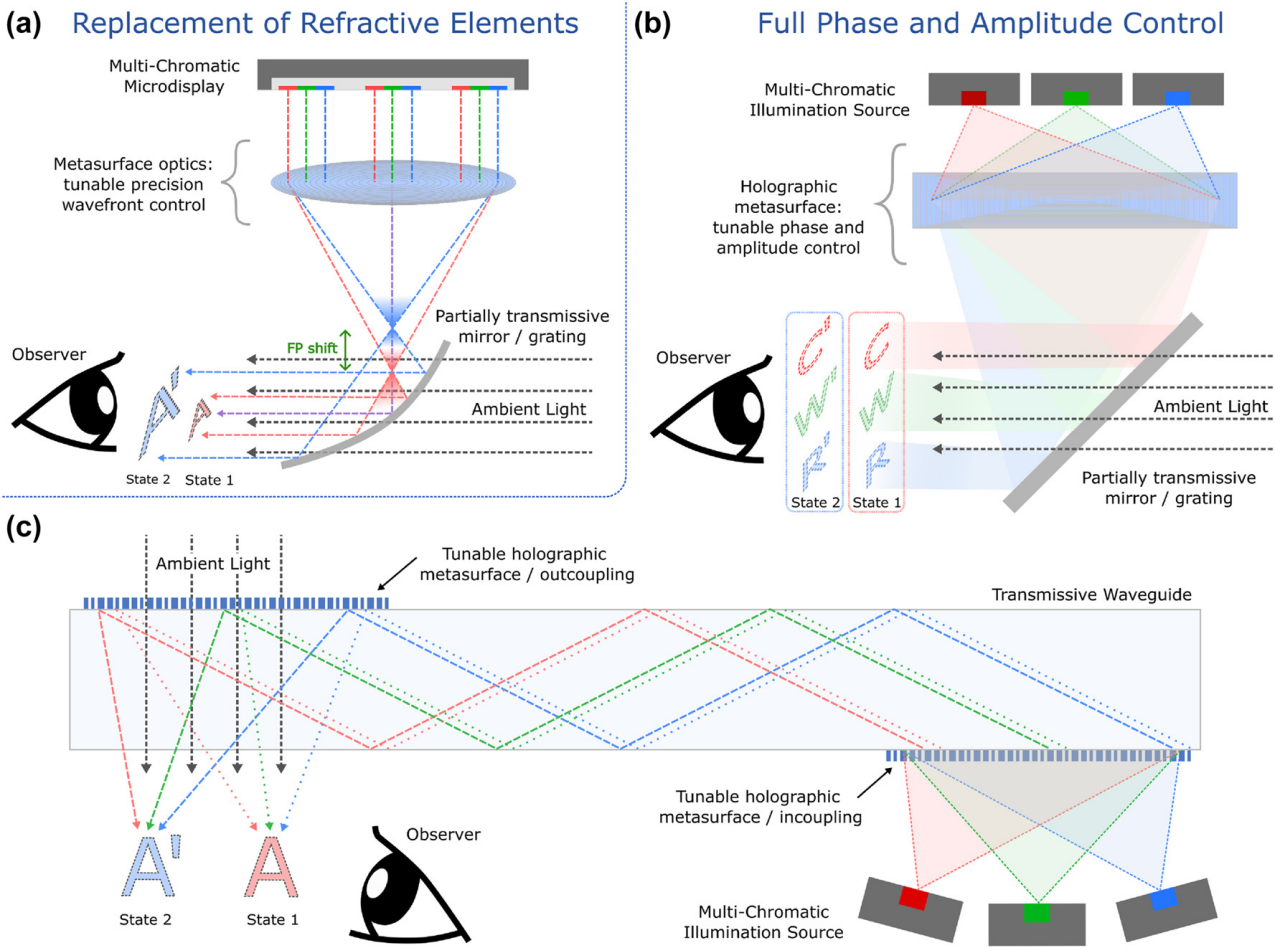
Avenues for integrating tunable metasurfaces with AR/VR devices. (a) Replacing traditional refractive elements with metasurface devices, a simplified optical schematic is shown with a tunable meta-lens. (b) Schematic implementation of highly reconfigurable holographic metasurfaces with full phase and amplitude control to replace image generation engines. (c) Schematic implementation of tunable metasurface technology providing both hologram generation and tunable input and output coupling in optical waveguides.

There are two major pathways for tunable metasurface integration in AR/VR devices: tunable discrete optical elements and highly tunable holographic metasurfaces. We propose that these pathways both offer significant improvements for current AR/VR devices, meeting the challenges outlined above. In the former case, elements such as lenses and collimators are replaced with their metasurface equivalents, enabling greater precision in wavefront control. These are demonstrated in Figure 4(a) and can include, for instance, higher quality lensing through metalens technology [14]. Tunability in the metasurface can - furher enable highly flexible optical systems, such as varifocal lensing. This approach builds on traditional metasurface technologies to enhance AR/VR devices in their current form. Several studies have been explored in this direction, and with meta-optics rapidly approaching the stage of commercial viability, the technology is poised and ready for practical implementation [84]. Metalens integration has the potential to meet many of the challenges with AR/VR devices outlined above.

Beyond drop-in replacement, tunable holographic metasurfaces have the potential to integrate the entire optical propagation pathway – including image generation, light propagation and steering, and projection – into a single device with small form-factor. As shown in Figure 4(b) and (c), the simplest approach uses a single surface to fully control both the propagation phase and amplitude. The optical pathway is simplified in this figure for clarity. A similar picture applies to waveguide AR/VR devices, where holographic metasurfaces can be integrated as direct surfaces or as tunable in- and out-coupling gratings for complete spatial light control. Metasurfaces have already demonstrated their effectiveness as replacements for diffractive elements, particularly in mitigating shadowing effects [85]. Although recent studies have made progress toward implementable devices, significant advancements are still required to improve both tunable metasurfaces and their application in AR/VR devices. However, the potential benefits of this approach are substantial, as these devices offer a pathway to achieving complete wavefront control, necessary for the next-generation of AR/VR devices.

### 4.1 Advancing tunable metasurfaces

Cost-effective and efficient fabrication strategies are critical for future metalens applications, given the widespread popularity of AR/VR devices and expected future market increases. These limitations make traditional lithography problematic. We propose nanoimprint lithography as a critical technique for future commercially viable applications. While the technique has been around many years, continual advancements in reproducibility and minimum feature size could drive further improvements [46], [86]. Design considerations must also account for the inherent limitations of the method in feature size and shape.

Although tunability on both global and local scales can be relevant for AR/VR applications, full wavefront control in a holographic metasurface will require a much greater degree of local control or addressability, ideally but not necessarily down to the single meta-element level. One promising approach for achieving this level of tunability is gated electronic tuning of excitonic 2D materials, such as transition metal dichalcogenides (TMDs). However, alternative strategies, such as the integration of liquid crystals and metasurfaces, also hold significant potential and deserve further investigation.

### 4.2 Optimization and design

As metasurface complexity advances toward free-form or low-correlation structures, the algorithmic demands for geometrical optimization becomes increasingly challenging. This is particularly true with the introduction of locally tunable configuration states. Poor optimization leads to undesirable results, including suboptimal devices and intentional limitations on the design parameter space. Finally, the computational power requirements for real-time metasurface reconfiguration in response to user stimuli must be considered. This processing is further constrained by the need to operate on a compact, wearable AR/VR device without excessive weight or heat generation.

New approaches to metasurface optimization and design are essential to fully optimize holographic metasurfaces for AR/VR applications. Machine learning stands out as a promising approach, though alternative approaches should not be overlooked [8], [87]. In particular, techniques such as physics-informed neural networks appear to be extremely promising, although more work is needed to effectively train large-scale networks [8], [50], [88], [89].

### 4.3 AR/VR integrations

As mentioned above, the AR/VR environment presents significant technical challenges for implementing tunable holographic metasurface technologies beyond standard metrics such as tunability and wavefront reconstruction quality. Key limitations include weight, form factor, heat generation, human compatibility and safety, cost-effectiveness, production scalability, and long-term reliability. These challenges arise from the need to position the device close to the human eye and support it with the head, as well as the difficulties of commercial scalability [35], rendering certain tunability approaches impractical. Addressing these issues will be paramount for the development of practical, commercially viable AR/VR metasurface devices.
